# Transcriptome Analyses Reveal Adult Metabolic Syndrome With Intrauterine Growth Restriction in Pig Models

**DOI:** 10.3389/fgene.2018.00291

**Published:** 2018-08-15

**Authors:** Linyuan Shen, Mailin Gan, Shunhua Zhang, Jideng Ma, Guoqing Tang, Yanzhi Jiang, Mingzhou Li, Jinyong Wang, Xuewei Li, Lianqiang Che, Li Zhu

**Affiliations:** ^1^College of Animal Science and Technology, Sichuan Agricultural University, Chengdu, China; ^2^Department of Cell and Developmental Biology, University of Illinois at Urbana–Champaign, Champaign, IL, United States; ^3^College of Life Science, Sichuan Agricultural University, Chengdu, China; ^4^Chongqing Academy of Animal Science, Chongqing, China; ^5^Institute of Animal Nutrition, Sichuan Agricultural University, Chengdu, China

**Keywords:** pig, mRNA, transcriptome, IUGR, liver, gluconeogenesis

## Abstract

Epidemiological data have indicated that intrauterine growth retardation (IUGR) is a risk factor for the adult metabolic syndrome in pigs. However, the causative genetic mechanism leading to the phenotype in adulthood has not been well characterized. In the present study, both normal and IUGR adult pigs were used as models to survey the differences in global gene expression in livers through transcriptome sequencing. The transcriptome libraries generated 104.54 gb of data. In normal and IUGR pigs, 16,948 and 17,078 genes were expressed, respectively. A total of 1,322 differentially expressed genes (DEGs) were identified. Enrichment analysis of the DEGs revealed that the top overrepresented gene ontology (GO) terms and pathways were related to oxidoreductase activity, ATPase activity, amino catabolic process, glucose metabolism, and insulin signaling pathway. The increased gluconeogenesis (GNG) and decreased glycogen synthesis in the liver contributed to the glucose intolerance observed in IUGR. The reduced expression of insulin signaling genes (such as *PI3K* and *AKT*) indicated an elevated risk of diabetes in adulthood. Together, these findings provide a comprehensive understanding of the molecular mechanisms of adult IUGR pigs and valuable information for future studies of therapeutic intervention in IUGR metabolic syndrome.

## Introduction

The period of intrauterine growth and development is one of the most vulnerable periods in mammalian lifetime. Unfortunately, impairments to the embryo/fetus or its organs during gestation result in intrauterine growth restriction (IUGR) (Verma and Chaudhary, 2016). In humans, about 23.8% of newborns and approximately 30 million babies worldwide suffer from IUGR every year (Harmayani et al., 2017). Previous studies have associated increased morbidity and mortality, delayed postnatal growth and development, and increased susceptibility to metabolic syndrome with IUGR early in life (Pallotto and Kilbride, 2006). As the number of cases with metabolic syndromes is rapidly increasing, fetuses and neonates with IUGR are strongly predisposed to obesity, diabetes, and cardiovascular disease in later life based on experimental animal models and human epidemiological data (McMillen and Robinson, 2005; Manten et al., 2007; Martin-Gronert and Ozanne, 2007). The liver is the most important organ in the regulations of numerous metabolic processes, including hormone production and glucose and lipid metabolism (Ramayo-Caldas et al., 2012). Until now, hepatic metabolism is the preferential model system through which mechanisms of IUGR are examined. For example, offsprings exposed to intrauterine malnutrition have an increased risk of developing abnormal glucose metabolism along with increased hepatic gluconeogenesis (Thorn et al., 2009, 2013; George et al., 2012) and reduced hepatic glycolysis (Bogdarina et al., 2004). Additionally, defective insulin signaling cascade pathway would be expected to impair glucose uptake in the muscle, which would induce overproduction of glucose in the liver, ultimately leading to the development of glucose intolerance (Bogdarina et al., 2004; Samuel and Shulman, 2012). It has also been reported that IUGR pigs showed increased fatty acid flux toward the liver and reduced lipolysis and fatty acid oxidation (Yan et al., 2017). Together, these changes can lead to lipotoxicity, further reducing insulin sensitivity in the liver (Karpe et al., 2011; Yan et al., 2017). Recently, hepatic mitochondrial dysfunction was demonstrated to be a potential mechanism of IUGR development. These studies reported that IUGR during infancy decreased the capacity of hepatic mitochondrial biogenesis and the metabolism efficiency of oxidative phosphorylation (OXPHOS), resulting in excess production of mitochondrial superoxide radicals and poor hepatic antioxidant defense systems (Che et al., 2015; Zhang et al., 2016, 2017).

Although there is a wealth of evidence to show that IUGR can increase the risk of adult metabolic diseases such as cardiovascular disease, obesity, and type 2 diabetes (Ross and Beall, 2008; Rinaudo and Wang, 2012), the genetic mechanisms underlying IUGR are not well characterized. At present, next-generation sequencing, a powerful method that can be used to understand complete gene regulatory networks relevant to IUGR, is being applied to analyze global transcriptomic changes (Mutz et al., 2013). Although there are some studies about transcriptome of IUGR organs (such as placenta, kidney, etc.) in human and mouse models (Tain et al., 2015; Ackerman et al., 2017), metabolic syndrome of IUGR induced by aberrant liver transcriptome is still not well characterized. Therefore, we used pigs as biomedical models for the study of the metabolic syndrome of IUGR in humans because of the similar morphology, physiology, metabolism, and proportional organ sizes between pigs and humans. Furthermore, pigs have the advantage of relatively low genetic and environmental variances, whereas humans live in a highly confounding environment, where personal lifestyle choices such as smoking and alcohol consumption have significant impacts on the incidence of metabolic syndrome (Li et al., 2012). The aim of this study was to employ pig hepatic transcriptome analyses to reveal the genetic mechanisms of metabolic syndrome arising from IUGR.

## Materials and Methods

### Ethics Statement

All the animal experimental and sample collection procedures were approved by the Institutional Animal Care and Use Committee of the College of Animal Science and Technology of Sichuan Agricultural University, Sichuan, China, under permit No. DKY-B20131403 (Ministry of Science and Technology, China, revised in June 2004). All experimental methods were performed in accordance with the Sichuan Agricultural University of Health Guide for the Care and Use of Laboratory Animals.

### Animal Housing and Sample Collection

A total of eight pairs of normal birth weight (∼1.49 kg) and IUGR (∼0.97 kg) PIC breeding male piglets chosen from eight sows were used in this study. One IUGR piglet and one control piglet were selected from each litter. All selected pigs were moved to nursing cages at 7 days of age. The animals were fed with milk formula until 28 days of age. The formula composition and nutrient levels of the milk formula fed to the pigs are provided in **Supplementary Table S1**. After 28 days, the composition of diets fed to pigs was adjusted according to body weight (**Supplementary Table S2**). In all stages, pigs had *ad libitum* access to feed and water and were housed in the same environment. At 150 days of age, all pigs were kept off feed but given free access to water for 24 h, then electrically stunned, exsanguinated, scalded, and rinsed. Serum was harvested by centrifugation at 3000 *g* for 10 min at 4°C and stored at -80°C. Other samples were obtained from the core of the liver and longissimus dorsi immediately after exsanguination and rapidly frozen in liquid nitrogen.

### Biochemical Parameters and Metabolite Measurement

The concentrations of glucose (No. F006), glucogen (No. A043), triglyceride (#A110-2), free fatty acid (No. A042-1), and malondialdehyde (MDA) (No. A003-1) were determined using commercial kits (Nanjing Jiancheng Institute of Bioengineering, Nanjing, Jiangsu, China) following the manufacturer’s instructions. The levels of hepatic adenosine triphosphate (ATP) and adenosine diphosphate (ADP) were measured using high-performance liquid chromatography (HPLC), following a previously published method (Zhang et al., 2017). The activity of ATPase (No. A070-2), cytoplasmic copper/zinc superoxide dismutase (CuZn SOD) (No. A062), and glutathione reductase (GR) was also determined using commercial kits (Nanjing Jiancheng Institute of Bioengineering, Nanjing, Jiangsu, China). Sample data were collected using an automatic biochemical analyzer (Model 7020, Hitachi, Tokyo, Japan) and a UV-1100 spectrophotometer (Shanghai Mapada Instruments Co. Ltd., Shanghai, China).

### Intravenous Glucose Tolerance Test

After overnight fasting, an intravenous glucose tolerance test (i.v.GTT) was conducted in all animals as previously described by Wang et al. (2016). Briefly, at 149 days of age, dextrose (500 g/L) was infused continuously through ear venipuncture over the course of 6 min. The infusion dose and rate were 0.5 g/kg of body weight and 10 g glucose/minute, respectively. Blood samples were obtained at -6, -4, -2, and 0 min relative to the completion of dextrose infusion and at 5, 10, 15, 30, 60, 90, and 120 min post administration. Blood glucose was measured using an Ascensia Elite glucometer (Bayer Healthcare Company, Leverkusen, Germany). All samples were measured in duplicate.

### Total Ribonucleic Acid (RNA) and Deoxyribonucleic Acid (DNA) Extraction

Total RNA was extracted from liver and muscle samples using TRIzol (Invitrogen, CA, United States). Nucleic acids were then further purified with RNeasy column (Qiagen, United States) according to the manufacturer’s protocol. RNA integrity and concentration were assessed using the Bioanalyzer 2100 (Agilent Technologies) and NanoDrop (Thermo Technologies), respectively. DNA was isolated from liver samples for the measurement of mitochondrial DNA (mtDNA) copy number using the DNeasy Blood & Tissue Kit (Qiagen, United States).

### Measurement of mtDNA Copy Number

The relative mtDNA copy number was determined by quantitative polymerase chain reaction (qPCR). The quantitative reverse transcription polymerase chain reaction (qRT-PCR) was performed using the SYBR Green Real-time PCR Master Mix (Takara, Dalian, China) on a CF96 Real-Time PCR Detection System (Bio-Rad, Richmond, CA, United States). The ratio of mitochondrial genes (ATP6 and COX2) to nuclear DNA single copy gene (GCG) within the same sample was used to calculate the mtDNA content (primer sequences are listed in **Supplementary Table S3**). All reactions were performed in triplicate. Relative mtDNA copy number per diploid cell was calculated by the 2^Δ^*^C^*^t^ method.

### Messenger RNA (mRNA) Library Construction and Sequencing

We randomly selected three pigs from each group for deep sequencing analysis. First, only RNA samples that had RNA Integrity Number (RIN) scores > 8 were used for sequencing. Approximately 5 μg of total RNA from a single liver tissue was isolated by binding of the Poly (A) tails on the mRNA with poly-T oligo conjugated magnetic beads (Thermo Fisher). Following purification, the mRNA was fragmented into small pieces using divalent cations under elevated temperature. The cleaved RNA fragments were then reverse transcribed into the final complementary DNA (cDNA) library in accordance with the Illumina RNA ligation-based method (Illumina, San Diego, CA, United States). The constructed RNA libraries were sequenced using an Illumina HiSeq 2000 platform. Paired-end reads, approximately 90 bp long, were generated.

### Transcriptome Data Analysis

Clean data were obtained by removing reads containing adapters, reads containing over 10% of poly(N), and low-quality reads (>50% of the bases had Phred quality scores ≤ 10) from the raw data. All the downstream analyses were conducted only on high-quality data. The index of the reference genome was built using Bowtie v2.0.6 and paired-end clean reads were aligned to the reference genome using TopHat v2.0.14. The mapped reads from each library were assembled with Cufflinks v2.2.1. The reference annotation-based transcript (RABT) assembly method in Cufflinks v2.2.1 was used to construct and identify mRNA transcripts from the TopHat2 alignment results. Cuffdiff v2.1.1 was used to calculate fragments per kilobase of exon model per million mapped reads (FPKM) scores for transcripts in each library. Differentially expressed genes (DEGs) were identified through pairwise comparisons between every two stages using edgeR (release 3.2). The statistical significance of gene expression differences was evaluated using an adjusted *P*-value (*Q*-value) < 0.01 and|log_2_(fold change)|≥ 2 as the threshold. In addition, the cluster of the DEGs was performed by using common perl and R scripts.

### Functional Enrichment Analysis

Differentially expressed genes were converted to human orthologous genes and submitted to the DAVID 6.8 web server^1^ for enrichment analysis of the significant overrepresentation of GO biological processes (GO-BP), molecular function (GO-MF) terminologies, and Kyoto Encyclopedia of Genes and Genomes (KEGG) pathway category. In all tests, the *P*-values were calculated using Benjamini-corrected modified Fisher’s exact test. Only *P*-values < 0.05 were considered as significant.

### Quantitative RT-PCR

The expression levels of selected genes were quantified using qRT-PCR. The qRT-PCR was performed using the SYBR Green Real-time PCR Master Mix (Takara, Dalian, China) on a CF96 real-time PCR detection system (Bio-Rad, Richmond, CA, United States). The PCR primer sequences used are presented in **Supplementary Table S3**. *ACTB, TBP*, and *TOP2B* genes were simultaneously used as internal control genes for normalization. All measurements contained a negative control (no cDNA template). Each RNA sample was analyzed in triplicate. The 2^-ΔΔ^*^C^*^t^ method was used to determine the relative abundance of each mRNA.

### Statistical Analyses

Data were analyzed with SPSS (21.0 version). All data were presented as means ± standard deviation (SD). Differences in groups were analyzed with Student’s *t*-test. *P* < 0.05 was considered to be statistically significant.

## Results and Discussion

### Phenotypic Traits of Adult IUGR Pig

**FIGURE 1:**
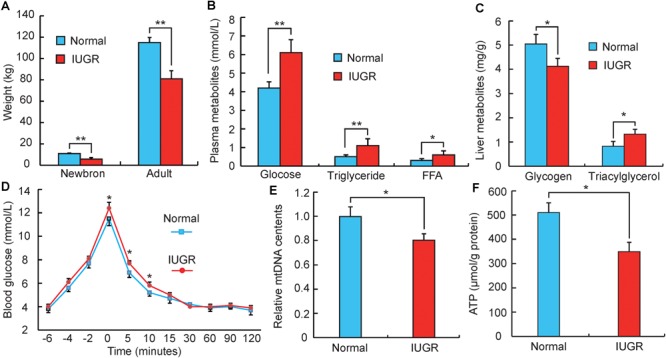
Phenotypic differences between adult normal and IUGR pigs. **(A)** The body weights of normal and IUGR pigs as newborns and adults (*n* = 8). **(B)** The blood glucose, triglyceride and free fatty acid (FFA) concentrations in adult normal and IUGR pigs (*n* = 8). **(C)** The contents of glycogen and triglyceride in livers of adult normal and IUGR pigs (*n* = 8). **(D)** Plasma glucose concentrations after an intravenous glucose tolerance test (i.v.GTT) at 149 days of age. Time indicates minutes relative to the completion of dextrose infusion (*n* = 8). **(E)** The copy number of hepatic mitochondrial DNA in adult normal and IUGR pigs (*n* = 8). **(F)** The contents of ATP in livers of adult normal and IUGR pigs (*n* = 8). Data are means ± SD. Statistical significance was calculated by Student’s *t*-test (*n* = 8 per individual). Significant differences levels: ^∗^*p* < 0.05, ^∗∗^*p* < 0.01.

According to a previous study (Che et al., 2010), pigs with birth weights within 0.5 standard deviations of the mean litter birth weight were considered to be normal, whereas those with lower weights (1.5 standard deviations below the mean litter weight) were defined as IUGR. As presented in **Figure 1A**, IUGR piglets had lower birth weights (32–39% decrease, *p* < 0.01) than normal piglets, and this difference persisted until adulthood (19–25% decrease, *p* < 0.01). This suggests that impaired intrauterine growth and development may have a persistent influence on metabolic efficiency later in life. It was observed that adult IUGR pigs exhibited greater plasma concentrations of glucose (*p* < 0.01) and lower hepatic glycogen (*p* < 0.05) (**Figures 1B,C**). Previous studies have reported that hyperglycemia phenotypes are typically associated with glucose intolerance and type 2 diabetes (Nathan et al., 2009). These findings were supported by our observations during the intravenous glucose tolerance test (**Figure 1D**). Blood glucose concentrations were higher at post-infusion times 5 (*p* < 0.05) and 10 min (*p* < 0.05) in IUGR pigs than normal group. Moreover, adult IUGR pigs also exhibited greater plasma concentrations of triglyceride and free fatty acids, as well as triglyceride content in livers (**Figures 1B,C**). These results imply that IUGR likely impairs the capacity of hepatic fatty acid oxidation, thus promoting the development of the hyperlipidemia phenotype. This is consistent with recent studies reporting that IUGR was the predominant factor in affecting lipid metabolism (Koklu et al., 2006; Li et al., 2016). Interestingly, the influence of high circulating triacylglycerol and non-esterified fatty acid was also demonstrated to promote glucose intolerance (Karpe et al., 2011; Lopez et al., 2011). Furthermore, OXPHOS was determined to be an important component of hepatic metabolism. This was consistent with other previous studies, which demonstrated that suckling piglets of IUGR impaired hepatic mitochondrial biogenesis and energy homeostasis (Zhang et al., 2017). As shown in **Figures 1E,F**, mtDNA and ATP were both significantly decreased in adult IUGR pigs (*p* < 0.05). This implies that the change of the OXPHOS pathway during gestation might persist well into adult life. Overall, these results suggest that adult IUGR pigs have multiple metabolic syndromes, and differences in the transcriptome were likely to underlie the aberrant phenotypes.

### Summary of Transcriptome Data

**FIGURE 2:**
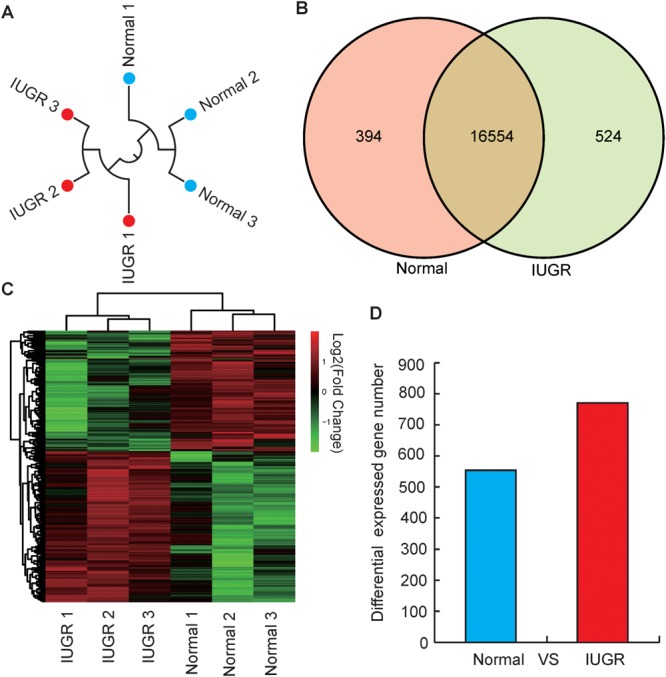
Genome-wide distribution of DGEs between normal and IUGR pigs. **(A)** Hierarchical clustering of samples using all expressed genes for biological reproducibility analysis. **(B)** Venn diagram of expressed genes between normal and IUGR pigs. **(C)** Heat map diagram of DGEs between normal and IUGR pigs **(D)** Distribution of DEGs between normal and IUGR pigs.

To identify genes involved in the differentially effective metabolic pathway between adult IUGR and normal pigs, RNA transcriptome libraries were constructed using adult pig liver tissues. Transcriptome libraries generated a total of 1,161 million pair-end reads of 90 bp in length. The total sequencing length was 104.54 gb, representing approximately 43× coverage of the complete pig genome. Approximately 64.30–68.68% of all reads were unique and aligned to the University of California, Santa Cruz (UCSC) pig reference genome (Sus scrofa 11.1) using the TopHat2 package (**Table 1**). Furthermore, 85.84–91.41% of all reads were located within exons (**Supplementary Figure S1**). In this study, if one transcript was expressed in all the three biological replicates of the constructed cDNA libraries, it was considered to be an expressed transcript and included for subsequent analysis. Consequently, 16,948 and 17,078 known transcripts were identified as being expressed in normal and IUGR pigs, respectively. Among the transcript populations, only a small number of genes were highly expressed (**Supplementary Tables S4, S5**). These findings were consistent with previous studies, which reported that only a small number of highly expressed genes may play cellular housekeeping gene roles and regulate cellular components and basal cellular metabolism (Shen et al., 2015). The reproducibility and reliability of transcriptome libraries were analyzed by total expressed genes using hierarchical clustering. As presented in **Figure 2A**, the three biological replicates of each group were highly correlated with one another (Pearson’s *r* > 0.96), and all the three libraries of each group were definitively assigned to a single cluster. This verified the high reproducibility and reliability of the transcriptome profiling performed in the present study. To explore the global transcriptional changes, we identified a total of 16,554 transcripts which were co-expressed in normal and IUGR pigs and identified 1322 DEGs (**Figure 2B** and **Supplementary Table S6**), including 558 highly expressed genes in the normal group and 764 highly expressed genes in the IUGR group (**Figures 2C,D**). The heat map of the hierarchical clustering analysis indicated that the DEG data were also highly reproducible (**Figure 2C**). Furthermore, the transcriptome sequencing results were also validated through the qRT-PCR analysis of expression patterns of eight randomly selected genes from each group. The results indicated that the expression patterns of these genes were highly consistent between the two methods (Pearson’s *r* > 0.81, *p* < 0.01; **Supplementary Figure S2**).

**Table 1:** Summary of transcriptome alignment.

Group	Normal-1	Normal-2	Normal-3	IUGR-1	IUGR-2	IUGR-3
Total raw reads	182462984	188671562	188490354	212157764	191055696	198755512
	(16.42G)	(16.98G)	(16.96G)	(19.09G)	(17.20G)	(17.89G)
Valid data	181246976	186904702	186844562	210590742	189842370	197354938
Valid ratio (reads)	0.99	0.99	0.99	0.99	0.99	0.99
Q20%	99.50	99.55	99.63	99.53	1.00	1.00
Total mapped ratio %	72.49	70.11	69.57	70.68	73.79	73.96
Unique mapped ratio %	66.90	64.32	64.30	65.61	68.56	68.68
GC content %	46.43	45.26	45.18	45.12	45.82	45.22

### Functional Enrichment Analysis for Differential Expression Genes

**FIGURE 3:**
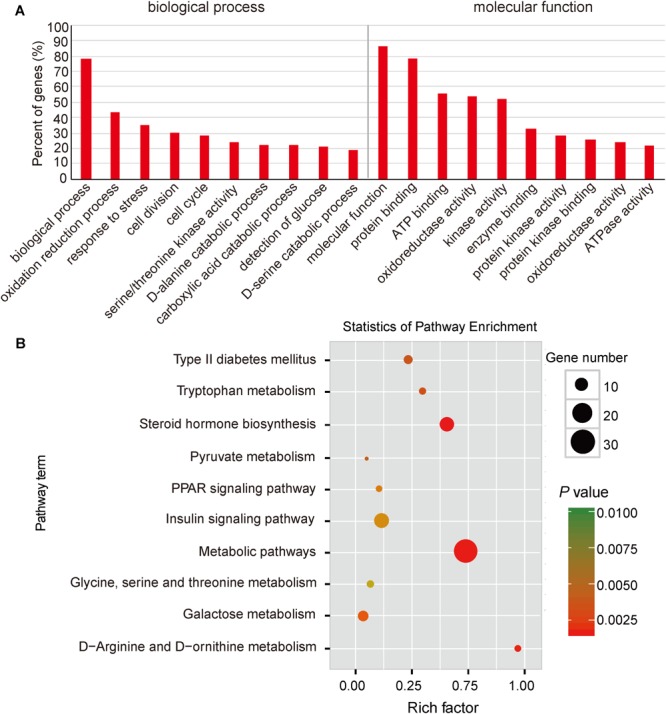
Functional enrichment analysis of DGEs between normal and IUGR pigs. **(A)** Gene Ontology (GO) categories enriched for DEGs between normal and IUGR pigs. **(B)** KEGG pathway enrichment analysis of DGEs between normal and IUGR pigs. The *P*-values were calculated using Benjamini-corrected modified Fisher’s exact test.

To explore the potential functions of DEGs, we performed a GO enrichment analysis. As shown in **Figure 3A**, the top significantly overrepresented GO terms were related to two main classes of biological functions: energy metabolism (including oxidation reduction process, oxidoreductase activity, ATP binding, and ATPase activity) and amino and glucose metabolism (including serine/threonine kinase activity, D-alanine and D-serine catabolic processes, and detection of glucose) (**Supplementary Table S7**). Mitochondrion is considered as the central organelle with respect to its functions in energy metabolisms and the major sites of ATP productions in the body (Hock and Kralli, 2009). Interestingly, previous studies have suggested that mitochondrial dysfunction is an underlying mechanism responsible for the IUGR. Mortensen et al. (2010) observed that mouse IUGR offsprings exhibited abnormal expression patterns of mitochondrial function-related genes in liver tissue. Also, in a juvenile pig model, it has been reported that post-weaning IUGR pigs exhibited impaired mitochondrial respiration functions, reduced mitochondrial DNA (mtDNA) contents and ATP productions, and impaired antioxidant defense systems in livers (Liu et al., 2012; Che et al., 2015; Zhang et al., 2017). Moreover, amino acids are one of the major sources of carbon for hepatic gluconeogenesis, such as alanine, serine, and threonine (Sandoval and Sols, 1974; Egan et al., 1983). Here, we observed that serine and threonine catabolic metabolisms were highlighted in the functional enrichment analysis, which was in accordance with a previous report, in which IUGR status was associated with up-regulated gluconeogenic gene expressions (*PEPCK, G6P*, and *PGC1*α), which resulted in increased hepatic gluconeogenic capacity in a fetal sheep model (Thorn et al., 2009). Furthermore, previous studies have reported that elevated hepatic gluconeogenesis would reduce glucose tolerance (Thorn et al., 2011). These findings are consistent with our observations reported in **Figure 1D**.

Here, we also performed a KEGG pathway enrichment analysis. As presented in **Figure 3B**, the top enriched pathways were related to amino acid metabolisms (such as tryptophan, glycine, arginine, and ornithine), which are consistent with the activated pathway of hepatic gluconeogenesis in IUGR animals (Thorn et al., 2009). In addition, we also observed that pyruvate served as the substrate for gluconeogenesis, and this metabolic pathway was significantly enriched in IUGR animals (Seubert and Huth, 1965). The highlighted insulin signaling pathway and the development of type 2 diabetes mellitus in animals suggest that diabetes may be a fetal adaptation to undernutrition and likely persists until adulthood (**Figure 3B** and **Supplementary Table S8**). These findings are consistent with a previous report in which glucose intolerance and diabetes in pigs born from IUGR conditions were associated with down-regulated expression of insulin signaling genes (*IR, PI3K*, and *AKT1/2*) in the liver (Wang et al., 2016). As shown in **Figure 3B**, the peroxisome proliferator-activated receptor (PPAR) signaling pathway was involved in the regulation of the AMPK-SIRT1-PGC1α-PPAR signaling cascade, which is responsible for mitochondrial biogenesis, fatty acid oxidation, and OXPHOS (Cheng and Ristow, 2013). Overall, the transcriptome data have indicated that IUGR has a strong impact on many metabolic pathways in adult pigs.

### Differential Expression of Genes Related to Glucose and Lipid Metabolism

**FIGURE 4:**
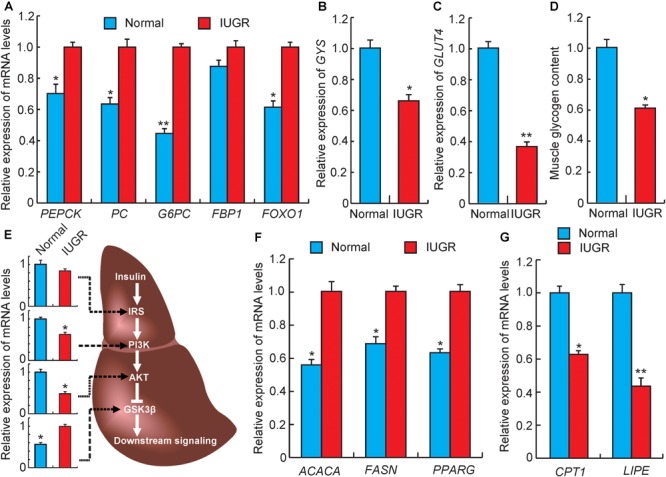
Gene expression related to glucose and lipid metabolism. **(A)** The expression levels of gluconeogenesis rate-limiting enzymes genes in livers. *PEPCK*, phosphoenolpyruvate carboxykinase; *PC*, pyruvate carboxylase; *G6PC*, glucose-6-phosphatase; FBP1, fructose-1,6-bisphosphatase 1; *FOXO1*, forkhead box O1. **(B)** The expression levels of glycogen synthesis gene (GYS) in livers. **(C)** The expression levels of glucose transporter gene (GLUT4) in muscles. **(D)** The glycogen levels in muscles. **(E)** The expression levels of genes related to insulin signaling in livers. *IRS*, insulin receptor substrate; *PI3K*, phosphatidylinositol 3-kinase; *AKT*, serine/threonine protein kinases; GSK3β, glycogen synthase kinase 3 beta. **(F)** The expression levels of fatty acid synthesis genes in livers. *ACACA*, acetyl-CoA carboxylase alpha; *FASN*, fatty acid synthase. **(G)** The expression levels of fatty acid oxidation genes in livers. *CPT1*, diacylglycerol cholinephosphotransferase; *LIPE*, lipase, hormone sensitive; *PPARG*, peroxisome proliferator activated receptor gamma. Data are means ± SD. Statistical significance was calculated by one-way repeated-measures analysis of variance (*n* = 3 per individual). ^∗^*p* < 0.05, ^∗∗^*p* < 0.01.

The excess accumulation of endogenous glucose played a crucial and direct role in the development of hyperglycemia and glucose intolerance. In the liver, the gluconeogenesis pathway is an important metabolic pathway for endogenous glucose synthesis, which is regulated by several important rate-limiting enzymes, such as PEPCK, G6PC, PC, and FBP1 (Wolfrum et al., 2003). Previous studies have demonstrated that increased gene expression of any of these enzymes would increase hepatic glucose production and plasma glucose levels in diabetics (Wolfrum et al., 2003; Collier and Scott, 2004). As presented in **Figure 4A**, the mRNA levels of *PEPCK* (*p* < 0.05), *G6PC* (*p* < 0.01), and *PC* (*p* < 0.05) were significantly up-regulated in liver tissues from IUGR pigs. In addition, *FOXO1*, a nuclear receptor relevant to glucose metabolism, was up-regulated in IUGR animals (**Figure 4A**). This results in the promotion of hepatic gluconeogenesis by directly binding to the *PEPCK* and *G6PC* promoters (Zhang et al., 2006; Jitrapakdee, 2012). Taken together, these results suggested that the capacity of hepatic gluconeogenesis is elevated in adult IUGR pigs. Glycogen synthesis in the liver is the alternate strategy through which endogenous glucose content is regulated. We observed that the expression of glycogen synthesis gene (*GYS*) was significantly decreased in IUGR pigs (**Figure 4B**), which was consistent with the observed glycogen levels in livers of IUGR pigs (**Figure 1C**). The skeletal muscle was another important tissue participating in the regulation of glucose metabolism, which could dispose of approximately 70–80% of postprandial glucose (Krook et al., 2000). *GLUT4* is considered to be the rate-limiting step of insulin-induced glucose uptake into the muscle (Krook et al., 2000). Previous studies have reported that defection of *GLUT4* could induce severe glucose tolerance (Kim et al., 2001), whereas overexpression of *GLUT4* would be expected to improve insulin sensitivity (Leturque et al., 1996). As expected, our results indicated that *GLUT4* expression in muscle tissue was significantly decreased in IUGR pigs (**Figure 4C**), which also agreed with the reduced glycogen content in muscle (**Figure 4D**). Overall, these outcomes contributed to hyperglycemia and glucose intolerance observed in adult IUGR pigs.

Insulin signaling is the major pathway to regulate glucose homeostasis, which is regulated by coordinating several important metabolic processes including glucose uptake, glycolysis, glucose oxidation, glycogen synthesis, and lipid synthesis and degradation in the liver (Saltiel and Kahn, 2001). The IRS–PI3K–AKT signaling cascade is the major insulin signaling pathway. Knockdown of expressions of those proteins would be expected to enhance gluconeogenesis and impair glycogen synthesis (Saltiel and Kahn, 2001). As expected, we observed that the mRNA levels of *PI3K* and *AKT* were decreased in livers from IUGR pigs (**Figure 4E**). Consequently, the decreased expression of *AKT* may further target *GSK3*β and activate its expression (Rommel et al., 2001), which would result in increased expression of gluconeogenic genes (such as *FOXO1*), accompanied by the promotion of gluconeogenesis and the impairment of glycogen synthesis (Sakamaki et al., 2012). This is similar to a previous study, which reported that maternal protein restriction would reduce the expression of insulin signaling proteins and lead to hyperinsulinemia in 21-month-old female rats (Fernandez-Twinn et al., 2005). Furthermore, insulin signaling was also involved in lipid metabolism, and a previous study has reported that the process of gluconeogenesis is correlated with increased fatty acid synthesis (Saltiel and Kahn, 2001). Nebendahl et al. (2013) reported that IUGR offsprings exhibited increased liver lipid contents than their littermates born with normal birth weights (Nebendahl et al., 2013). We also observed that IUGR pigs have higher triglyceride levels in livers than normal pigs (**Figure 1C**). The fatty acid synthesis genes (*ACACA* and *FASN*) and fat deposition gene (*PPAR*γ) were also observed to be up-regulated in livers of IUGR pigs (**Figure 4F**). However, other studies have demonstrated that increased lipogenesis in non-adipose tissues of IUGR offsprings may be attributed to impaired fatty acid oxidation capacity. Therefore, to further explore the mechanism of fatty acid oxidation in IUGR, we assessed expression patterns of genes related to lipolysis. *CPT-1* is the rate-limiting enzyme that determines fatty acid oxidation, and *LIPE* cleaves fatty acids from intracellular triglycerides for oxidation and export (Snel et al., 2012). As shown in **Figure 4G**, the expression levels of *CPT-1* and *LIPE* were down-regulated in adult IUGR livers, suggesting that the capacity of fatty acid oxidation was decreased in livers of IUGR pigs.

### Differential Expression of Genes Related to Mitochondrial Biogenesis and Energy Metabolism

**FIGURE 5:**
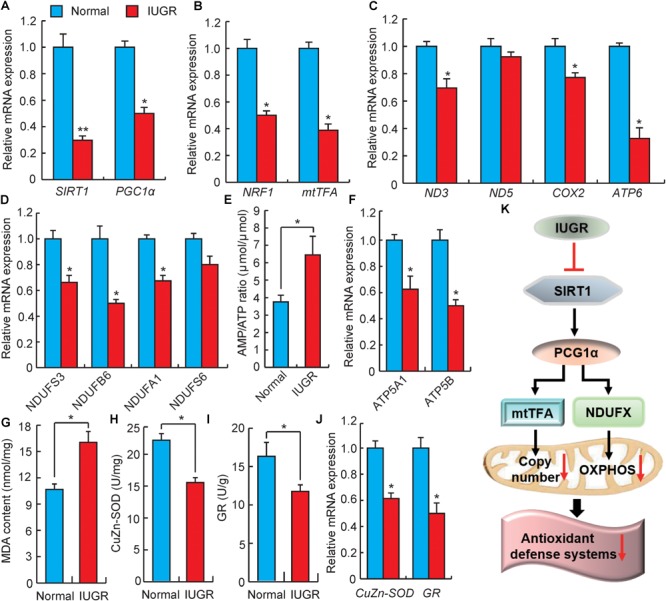
Analysis of genes involved in mitochondrial biogenesis and energy metabolism. **(A)** DEGs involved in energy homeostasis. *SIRT1*, sirtuin 1; *PGC1*α, PPAR coactivator 1 alpha. **(B)** Expression levels of mitochondrial biogenesis related genes. *NRF1*, nuclear respiratory factor 1; *mtTFA*, transcription factor A, mitochondrial. **(C)** The relative expression levels of mitochondrial genomic genes. *ND3*, NADH dehydrogenase subunit 3; *ND5*, NADH dehydrogenase subunit 5; *COX2*, cytochrome c oxidase subunit II; *ATP6*, ATP synthase F0 subunit 6. **(D)** The expression levels of genes associated with the electron transport chain. *NDUF*, NADH:ubiquinone oxidoreductase subunit. **(E)** The ratio of AMP/ATP in livers. **(F)** The expression levels of ATP synthesis genes. *ATP5A1*, ATP synthase, H+ transporting, mitochondrial F1 complex, alpha subunit 1; *ATP5B*, ATP synthase, H+ transporting, mitochondrial F1 complex, beta polypeptide. **(G)** The contents of malondialdehyde (MDA) in livers. **(H)** The activity of CuZn-SOD in livers. **(I)** The activity of GR in livers. **(J)** The relative expressions of CuZn-SOD and GR genes. *CuZn-SOD*, Superoxide dismutase 1; *GR*, glutathione reductase. **(K)** Possible pathways of IUGR mediation of hepatic energy metabolism and oxidative stress in adulthood. Data are means ± SD. Statistical significance was calculated by one-way repeated-measures analysis of variance (*n* = 3 per individual). ^∗^*p* < 0.05, ^∗∗^*p* < 0.01.

At present, IUGR suppression of mitochondrial function has been demonstrated in suckling pigs (Zhang et al., 2016, 2017), but the mechanisms resulting in this phenomenon in adults remain to be thoroughly characterized. *SIRT1* is considered to be a sensor of energy metabolism, which physically interacts with deacetylated *PGC1*α at multiple lysine sites (Cantó and Auwerx, 2009). Increasing *SIRT1* activity could modulate *PGC1*α functions *in vivo* and ultimately modulates the regulation of energy homeostasis (Lagouge et al., 2006). As shown in **Figure 5A**, the expressions of *SIRT1* and *PGC1*α were significantly down-regulated in livers of adult IUGR pigs. *PGC1*α is the central regulatory factor mediating mitochondrial biogenesis and OXPHOS to promote metabolisms (LeBleu et al., 2014). To confirm the decrease in mitochondrial biogenesis in adult IUGR pigs through the *SIRT1*/*PGC1*α pathway, we assessed the activity of the PGC1α/NRF1/mtTFA pathway. It was observed that the expression of *NRF1* and *mtTFA* were both decreased in IUGR livers (**Figure 5B**). It has been well established that *NRF1* and *mtTFA* transactivate the promoters of a number of mitochondrial-related genes and up-regulate mtDNA contents (May-Panloup et al., 2005). To further verify the decrease in mtDNA in adult IUGR pigs (**Figure 1E**), we measured randomly selected mitochondrial coding genes (Shen et al., 2016). As presented in **Figure 5C**, the expressions of *ND3, COX2*, and *ATP6* mRNAs were significantly decreased in livers of adult IUGR pigs (*p* < 0.05). These results suggest that the capacity of mitochondrial biogenesis is diminished in livers of adult IUGR pigs. The major mitochondrial function was affecting the efficiency of the electron movement through the electron transport chain and its coupling to OXPHOS to produce ATP. Therefore, to explore whether damaged mitochondrial biogenesis influenced the process of OXPHOS, we assessed the expressions of genes related to electron transport chain complexes (Pospisilik et al., 2007). We observed that most of these genes were down-regulated in IUGR livers (**Figure 5D**). The low efficiency of the electron transport chain in IUGR pigs resulted in decreased ATP production, which could be inferred through the increase in the AMP/ATP ratio (**Figure 5E**). The observed effect is consistent with the decreased expressions of ATP synthesis genes (*ATP5A1* and *ATP5B*) in livers of adult IUGR pigs (**Figure 5F**). These findings are corroborated by a previous report that the capacity of hepatic mitochondrial biogenesis and energy metabolism is decreased in weanling pigs (Su et al., 2017). All these results implied that IUGR would damage hepatic mitochondrial biogenesis and OXPHOS in adult pigs.

Previous studies have reported that dysfunctional mitochondria in IUGR can increase superoxide radical metabolites, which may lead to the development of oxidative stress (Biri et al., 2007). MDA would then accumulate in mitochondria, whereas the production of superoxide radicals increased in mitochondria (Che et al., 2015). The high content of MDA in IUGR livers suggested that adult IUGR livers produced excessive oxidative species (**Figure 5G**). This is in agreement with a previous report in which the antioxidative abilities in neonatal and adult IUGR mice livers were damaged (Gao et al., 2016). Interestingly, CuZn-SOD and GR were regarded as antioxidant enzymes, which would eliminate reactive oxygen species (ROS) (Yang et al., 2013). Here, the reduced activity of CuZn-SOD and GR in livers of adult IUGR pigs suggested the excessive production of free radicals, exhausting the antioxidant enzymes within the cells (**Figures 5H,I**). This was consistent with the reduced expressions of *CuZn-SOD* and *GR* mRNA in the IUGR animals (**Figure 5J**). These data suggested that the adult IUGR body possessed an oxidative status in the liver. This observation is consistent with the findings of a previous report, which showed that adult growth-restricted offsprings would exhibit increased oxidative stress and decreased antioxidant activity in a mouse model (Ojeda et al., 2012). Therefore, all these data indicate a possible mechanism of IUGR influenced hepatic energy metabolism and oxidative stress in adults, as presented in **Figure 5K**.

## Conclusion

In summary, this study provided a transcriptomic analysis between adult normal and IUGR pigs. We identified numerous DEGs, which are potentially associated with oxidoreductase activity, ATPase activity, amino catabolic processes, type 2 diabetes mellitus, and insulin signaling pathways. The expression pattern of DEGs suggested that adult IUGR pigs exhibited strong gluconeogenic activities but a low capacity for fatty acid oxidation, mitochondrial biogenesis, and OXPHOS. This study should serve as a valuable resource for further study of IUGR metabolic syndrome.

## Accession Codes

All the high-throughput sequencing data have been deposited in NCBI’s Gene Expression Omnibus under GEO Series accession numbers GSE106512.

## Author Contributions

LS, SZ, and LZ conceived and designed the experiments and drafted the manuscript. ML and LS performed the data analysis. SZ, JM, YJ, ML, JW, XL, LC, and GT collected the samples, performed the statistical analysis, and prepared nucleic acids. All authors read and approved the final manuscript.

## Conflict of Interest Statement

The authors declare that the research was conducted in the absence of any commercial or financial relationships that could be construed as a potential conflict of interest.

## Footnotes

1 https://david.ncifcrf.gov/
