# Supplementary material for: Transcriptome Analyses Reveal Adult Metabolic Syndrome With Intrauterine Growth Restriction in Pig Models
Source: Front Genet. 2018 Aug 15;9:291. doi: 10.3389/fgene.2018.00291 (PMC6103486; doi:10.3389/fgene.2018.00291)
Supplement: Supplementary file 1 [file Data_Sheet_1.DOCX]

Supplementary Material

Transcriptome analyses reveal metabolic syndrome of adulthood with intrauterine growth restriction in a pig model

Linyuan Shen, Mailin Gan, Shunhua Zhang, Jideng Ma, Guoqing Tang, Yanzhi Jiang, Mingzhou Li, Jinyong Wang, Xuewei Li, Lianqiang Che, & Li Zhu

*** Correspondence:** Lianqiang Che, email: [clianqiang@hotmail.com](mailto:clianqiang@hotmail.com)

Li Zhu, [zhuli7508@163.com](mailto:zhuli7508@163.com)

**Supplementary Table S1. Fornula milk**

| Ingredients | Percent(%) |
| --- | --- |
| Whole milk powder | 58.00 |
| Whey protein concentrate | 25.00 |
| Casein | 5.70 |
| Coconut oil | 10.00 |
| CaH2PO4 (22%P) | 0.10 |
| Choline chloride | 0.10 |
| Vitamin mixture^1^ | 0.10 |
| Mineral mixture^2^ | 0.50 |
| L-Arg | 0.06 |
| DL-Met | 0.06 |
| L-Lys.HCl | 0.30 |
| L-Thr | 0.03 |
| L-Trp | 0.05 |
| Total | 100.00 |
| Nutrient content^3^ | Content |
| Crude protein, % | 25.30 |
| Digestible energy, MJ/kg | 18.39 |
| Calcium, % | 1.02 |
| Total phosphorus, % | 0.81 |
| Available phosphorus, % | 0.67 |
| Digestive-Lysine, % | 1.93 |
| Digestive -Methionine, % | 0.63 |
| Digestive -Arginine, % | 0.86 |

Note 1: Contents per kg of diet: vitamin A, 0.94 mg; vitamin E, 20 mg; vitamin D3,10 μg; vitamin B1, 1.50 mg; vitamin B2, 5 mg; vitamin B6, 2 mg; vitamin B12, 40 μg; vitamin K3, 1 mg; folic acid, 1.50 mg; nicottinic acid, 20 mg; pantothenic acid, 15 mg; biotin, 0.1 mg. Note 2: contents per kg of diet: Zn, 90 mg; Mn, 4.0 mg; Fe, 90 mg; Cu, 6.0 mg; Se, 0.3 mg; I, 0.2 mg. Note 3: Nutrient levels were calculated values.

**Supplementary Table S2. Ingredient composition and nutrient levels of expreimental diets (as fed basis,%)**

| **Ingredients** | **7-11kg** | **11-25kg** | **25-50kg** | **50-75kg** | **75-100kg** |
| --- | --- | --- | --- | --- | --- |
| Corn grain | 27.19 | 35.00 | 68.57 | 75.13 | 78.03 |
| Expand corn | 25.00 | 23.68 | 0.00 | 0.00 | 0.00 |
| Soybean oil | 2.50 | 1.80 | 3.00 | 3.00 | 3.00 |
| Sucrose | 2.00 | 2.00 | 2.00 | 0.00 | 0.00 |
| Wheat bran | 0.00 | 0.00 | 3.00 | 3.00 | 5.00 |
| Whey powder | 12.00 | 7.00 | 0.00 | 0.00 | 0.00 |
| Soybean meal | 8.00 | 12.00 | 18.20 | 15.50 | 10.90 |
| Full-fat soybean | 5.00 | 8.00 | 0.00 | 0.00 | 0.00 |
| Soy protein concentrate | 5.00 | 3.00 | 0.00 | 0.00 | 0.00 |
| Whole milk powder | 3.00 | 0.00 | 0.00 | 0.00 | 0.00 |
| Fishmeal (CP 62.5 %) | 4.00 | 3.00 | 2.00 | 0.00 | 0.00 |
| Plasma protein powder | 3.00 | 1.00 | 0.00 | 0.00 | 0.00 |
| L-Lys·Hcl | 0.45 | 0.46 | 0.42 | 0.44 | 0.40 |
| DL-Met | 0.15 | 0.16 | 0.14 | 0.12 | 0.10 |
| L-Thr | 0.15 | 0.16 | 0.14 | 0.15 | 0.14 |
| L-Try | 0.01 | 0.02 | 0.03 | 0.03 | 0.03 |
| Choline chloride 50% | 0.15 | 0.10 | 0.10 | 0.10 | 0.10 |
| CaCO3 | 1.00 | 0.92 | 0.90 | 0.89 | 0.80 |
| CaHPO4 | 0.10 | 0.40 | 0.70 | 0.84 | 0.70 |
| NaCl | 0.30 | 0.30 | 0.30 | 0.30 | 0.30 |
| Premix^1^ | 1.00 | 1.00 | 0.50 | 0.50 | 0.50 |
| Total | 100.00 | 100.00 | 100.00 | 100.00 | 100.00 |
| **Nutrient levels^2^** |  |  |  |  |  |
| DE（Kcal/kg） | 3.58 | 3.48 | 3.39 | 3.40 | 3.39 |
| CP（%） | 19.55 | 18.35 | 15.68 | 13.73 | 12.18 |
| Ca（%） | 0.79 | 0.71 | 0.66 | 0.59 | 0.52 |
| STTD P（%） | 0.39 | 0.34 | 0.31 | 0.27 | 0.25 |
| SID Lys（%） | 1.36 | 1.23 | 0.98 | 0.85 | 0.73 |
| SID Met+cys（%） | 0.74 | 0.69 | 0.55 | 0.55 | 0.42 |
| SID Thr（%） | 0.80 | 0.73 | 0.58 | 0.52 | 0.46 |
| SID Trp（%） | 0.22 | 0.20 | 0.17 | 0.15 | 0.13 |

Note 1: Contents per kg of diet for 7-25 kg BW Period: Fe, 150 mg; Cu, 195 mg; Zn, 150 mg; Mn, 30 mg; I, 0.3 mg; Se, 0.3 mg; vitamin A, 12000 IU; vitamin D, 3200 IU; vitamin E, 80 mg; vitamin K3, 32.50 mg; vitamin B1, 2.30 mg; vitamin B2, 6.50 mg; vitamin B6, 5 mg; vitamin B12, 50 μg; nicotinic acid, 45 mg; pantothenic acid, 20 mg, folic acid, 1.50 mg; biotin, 0.15 mg; enzyme preparation and preservatives. Contents per kg of diet for 7-25 kg BW Period: Fe, 120 mg; Cu, 17 mg; Zn, 100 mg; Mn, 25 mg; I, 0.3 mg; Se, 0.2 mg; vitamin A, 5512 IU; vitamin D, 2250 IU; vitamin E, 24 mg; vitamin K3, 3 mg; vitamin B2, 6 mg; vitamin B6, 3 mg; vitamin B12, 24 μg;pantothenic acid, 15 mg, folic acid, 1.20 mg; biotin, 0.15 mg; enzyme preparation and preservatives. Note 2: Nutrient levels were calculated values.

**Supplementary Table S3. Information of primers used to perfrom Q-PCR**

| Gene symbol | Forword primer | Reverse primer |
| --- | --- | --- |
| *ATP6* | AAGCAAGGGTTCTCAAACTCG | CGGCGCAAACTAAACCAACTC |
| *COX2* | GCACCCCGACATAGAGAGC | CTGCGGAGTGCAGTGTTCT |
| *GCG* | GAATCAACACCATCGGTCAAAT | CTCCACCCATAGAATGCCCAGT |
| *HSD17B2* | TCTTCTCGGTGTCATGCTTCC | CAAAACTCCGGCAAATACCGT |
| *IGFBP2* | GACAATGGCGATGACCACTCA | CAGCTCCTTCATACCCGACTT |
| *HGD* | CCCGTCGTCATGGATCTCAC | GCTGACAATCTCCCTGTGGA |
| *NNMT* | GAGATCGTCGTCACTGACTACT | CACACACATAGGTCACCACTG |
| *DAO* | AATCTCGGGCTACAACCTCTT | TCAGCTTCCGAAATCCCAGAA |
| *GNMT* | CTGGGGTGGACTCCATTATGC | GATGACCCACTTGTCGAAGGC |
| *FOXO1* | GCGTGCCCTACTTCAAGGATAA | TCCCACTCTTGCCTCCCTCT |
| *GSR* | TGACTATGGCTTTCAGAGTTGTG | GCCGTGGATGATTTCTATGTG |
| *TOP2A* | CGAAATGACAAGCGAGAAGT | CTGACCAATGGGCTGTAAGA |
| *TCN1* | TCAGAGGGAACTATTCAATCA | GTCAGAGCAAGAACAGCCAC |
| *KCNIP3* | CTTCCTGTTCAATGCCTTCG | TAGCCATCCTTGTTAATGTCGTAG |
| *HAMP* | TCTCCCATCCCAGACAAGACAG | GACAGCAGCCGCAGCAGAAG |
| *ACTB* | TCTGGCACCACACCTTCT | TGATCTGGGTCATCTTCTCAC |
| *TBP* | GATGGACGTTCGGTTTAGG | AGCAGCACAGTACGAGCAA |
| *TOP2B* | AACTGGATGATGCTAATGATGCT | TGGAAAAACTCCGTATCTGTCTC |

*ATP6*: ATP synthase F0 subunit 6; *COX2*: cytochrome c oxidase subunit I; *GCG*: glucagon; *HSD17B2*: hydroxysteroid 17-beta dehydrogenase 2; *IGFBP2*: insulin like growth factor binding protein 2; *HGD*: homogentisate 1,2-dioxygenase; *NNMT*: nicotinamide N-methyltransferase; *DAO*: D-amino acid oxidase; *GNMT*: glycine N-methyltransferase; *FOXO1:* forkhead box O1; *GSR:* glutathione-disulfide reductase; *TOP2A:*  DNA topoisomerase II alpha; *TCN1*: transcobalamin 1; *KCNIP3:* potassium voltage-gated channel interacting protein 3; *HAMP:* hepcidin antimicrobial peptide; *ACTB*: actin beta; *TBP*: TATA-box binding protein; *TOP2B*: DNA topoisomerase II beta.

**Supplementary Table S4. Distuibutuion of genes expression (FPKM)**

| Sample | 0-0.1 | 0.1-0.3 | 0.3-3.57 | 3.57-15 | 15-60 | >60 |
| --- | --- | --- | --- | --- | --- | --- |
| Normal-1 | 18183  (15.02%) | 17054  (14.09%) | 65447  (54.06%) | 12987  (10.73%) | 4897  (4.05%) | 2487  (2.05%) |
| Normal-2 | 15005  (12.40%) | 14286  (11.80%) | 71180  (58.80%) | 14207  (11.74%) | 4325  (3.57%) | 2052  (1.70%) |
| Normal-3 | 14361  (11.86%) | 13047  (10.78%) | 73879  (61.03%) | 13879  (11.47%) | 3989  (3.30%) | 1900  (1.57%) |
| IUGR-1 | 15329  (12.66%) | 14830  (12.25%) | 70836  (58.52%) | 13462  (11.12%) | 4455  (3.68%) | 2143  (1.77%) |
| IUGR-2 | 17844  (14.74%) | 17732  (14.65%) | 65521  (54.12%) | 12808  (10.58%) | 4766  (3.94%) | 2384  (1.97%) |
| IUGR-3 | 17283  (14.28%) | 16802  (13.88%) | 66627  (55.04%) | 13383  (11.06%) | 4614  (3.81%) | 2346  (1.94%) |

Note, FPKM: Reads Per Kilobase of exon model per Million mapped reads


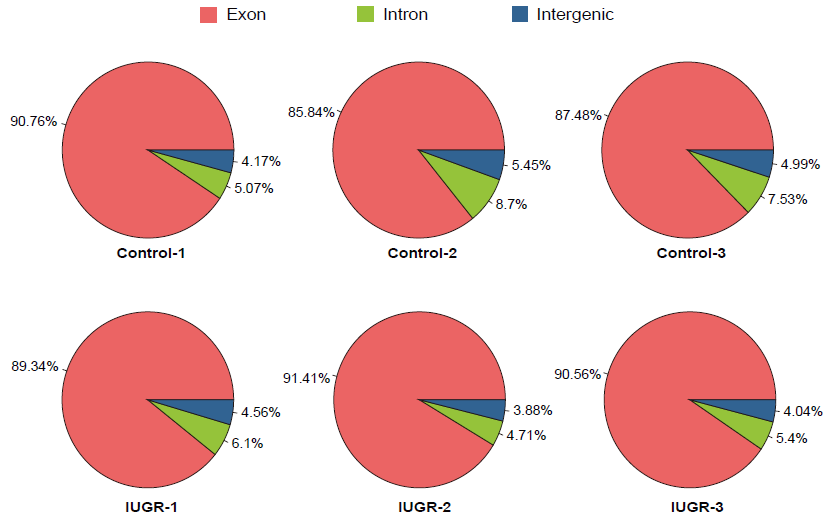


**Supplementary Figure S1. The distribution of reads in genome sites.**


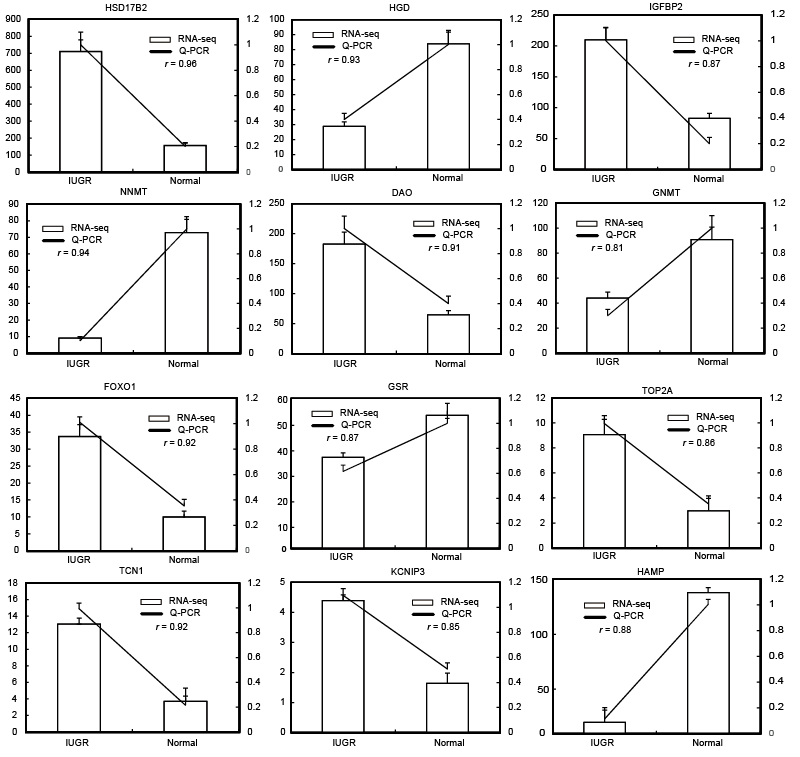


**Supplementary Figure S2. Validation of DEGs by qPCR. The *r* value represents the Pearson correlation coefficient between two methods**
